# Efficacy and safety of total glucosides of paeony in the treatment of systemic lupus erythematosus: A systematic review and meta-analysis

**DOI:** 10.3389/fphar.2022.932874

**Published:** 2022-12-07

**Authors:** Xiaohong Gong, Huan Li, Hongtao Guo, Shangwen Wu, Chaoqun Lu, Yiming Chen, Songwei Li

**Affiliations:** ^1^ Henan University of Chinese Medicine, Zhengzhou, China; ^2^ The First Affiliated Hospital of Henan University of Chinese Medicine, Zhengzhou, China; ^3^ Henan Provincial Hospital of Traditional Chinese Medicine, The Second Affiliated Hospital of Henan University of Chinese Medicine, Zhengzhou, China

**Keywords:** total glucosides of paeony, meta-analysis, safety, efficacy, systemic lupus erythematosus (SLE)

## Abstract

**Background:** Total glucosides of paeony (TGP), extracted from the Chinese medicine *Paeonia lactiflora* Pall., have been proven to be effective in various autoimmune diseases. We aim to systematically evaluate the efficacy and safety of TGP combined with different conventional therapeutic agents in the treatment of systemic lupus erythematosus (SLE).

**Methods:** Eight databases were searched for randomized controlled studies of TGP for SLE. The search time was set from the establishment of the databases to March 2022. The risk of bias was assessed by the Cochrane Evaluation Manual (5.1.0), RevMan 5.3 software was used for meta-analysis, and the certainty of the evidence was assessed by the GRADE methodology.

**Results:** A total of 23 articles were included, including 792 patients overall in the treatment group and 781 patients overall in the control group. The meta-analysis results showed that TGP combined with conventional treatments was superior to the conventional treatments in reducing the SLE disease activity and the incidence of adverse reactions (SMD_TGP+GC+CTX_ = −1.98, 95% Cl = [−2.50, −1.46], *p* < 0.001; SMD_TGP+GC+HCQ_ = −0.65, 95% Cl = [−1.04, −0.26], *p* <0.001; SMD_TGP+GC+TAC_ = −0.94, 95% Cl = [−1.53, -0.34], *p* < 0.05; SMD_TGP+GC_ = −1.00, 95% Cl = [−1.64, −0.36], *p* < 0.05; and RR_TGP+GC+CTX_ = 0.37, 95% Cl = [0.21, 0.64], *p* < 0.001). The results also showed that TGP helped improve other outcomes related to SLE disease activity, such as complement proteins (C3 and C4), immunoglobulins (IgA, IgM and, IgG), ESR, CRP, 24 h urine protein, and recurrence rate. In addition, TGP may also be effective in reducing the average daily dosage of glucocorticoids (GCs) and the cumulative dosage of cyclophosphamide (CTX). The certainty of the evidence was assessed as moderate to low.

**Conclusion:** TGP is more effective and safer when used in combination with different conventional therapeutic agents. It helped reduce the disease activity of SLE and the incidence of adverse reactions. However, we should be cautious about these conclusions as the quality of the evidence is poor. Future studies should focus on improving the methodology. High-quality randomized controlled trials (RCTs) will be necessary to provide strong evidence for the efficacy of TGP for SLE.

**Systematic Review Registration:** https://www.crd.york.ac.uk/PROSPERO, identifier CRD42021272481

## 1 Introduction

Systemic lupus erythematosus (SLE) is an autoimmune disease with multi-organ involvement, recurrent relapses and remissions, and the presence of a large number of autoantibodies in the body as the main clinical features, which can cause irreversible damage to the involved organs and eventually lead to the death of the patients if left untreated (Durcan et al., 2019; Fava and Petri, 2019). It is reported the global prevalence of SLE is about 0–241/100,000, and the prevalence of SLE in China is about 30–100/100,000, ranking second in the world (Li et al., 2013; Rees et al., 2017; Chinese Rheumatology Association, 2020). With the development of gene and molecular biology technology, the research on the pathogenesis diagnosis and treatment of SLE has made rapid progress. Although the 10-year survival rate for patients with SLE improved significantly from 63.2% in the 1950s to 91.4% in 2000s (Mu et al., 2018), the all-cause and cause-specific mortality rates remain significantly higher than the general population (Jorge et al., 2018; Bultink et al., 2021).

Drugs used in the treatment of SLE include glucocorticoids (GCs), hydroxychloroquine (HCQ), immunosuppressive (IS) drugs, and biological agents such as belimumab and rituximab (RTX). (Fanouriakis et al., 2019). However, long-term HCQ therapy can lead to retinal toxicity, with the incidence of retinal abnormalities exceeding 10% after 20 years of consecutive use (Knight et al., 2016; Kao et al., 2022). A long-term GC treatment can cause irreversible organ damage (Chen et al., 2018; Kwon et al., 2018). Combining IS drugs facilitates more rapid GC reduction and may prevent disease recurrence. However, the teratogenic potential of methotrexate (MTX) and azathioprine (AZA) and the toxic effect of cyclophosphamide (CTX) on the gonads have limited their widespread application in women and men of reproductive age (Knight et al., 2016; Martins et al., 2017; Tamirou et al., 2017). RTX and belimumab are usually considered following the failure of first-line therapies or relapsing disease (Iaccarino et al., 2015; Olfat et al., 2015; Fanouriakis et al., 2019; Huang et al., 2022). However, the price and potential risk of infection pose a huge financial burden and concern for patients (Steiger et al., 2022). Therefore, a safer and more effective therapeutic strategy needs to be explored.

**FIGURE 1:**
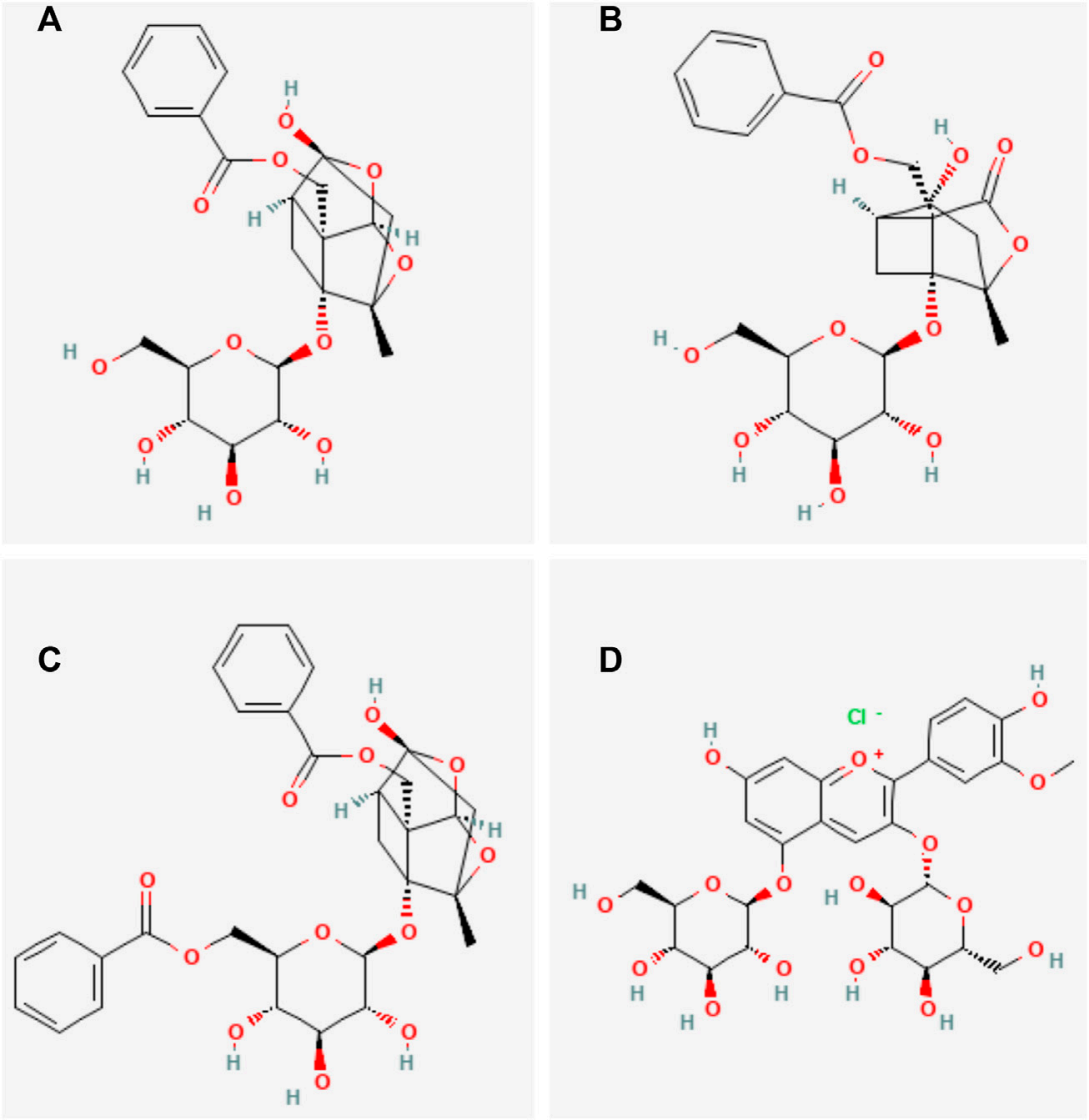
Main chemical structures of TGP. **(A)** Paeoniflorin, **(B)** Albiflorin, **(C)** benzoylpaeoniflorin, **(D)** Paeonin.

Total glucosides of paeony (TGP) are a group of active glycosides extracted from the roots of *Paeonia lactiflora* Pall. (Bai shao in Chinese), which mainly include paeoniflorin, paeonin, albiflorin, and benzoylpaeoniflorin (Figure 1). Paeoniflorin is the major active component of TGP. It constitutes more than 40% of TGP (Yang et al., 2021). Research studies have shown that TGP has analgesic, anti-inflammatory, immunomodulatory, and antioxidant functions (He and Dai, 2011; Zhang and Wei, 2020). TGP is often used as an adjunctive therapy for autoimmune diseases. It has been successfully utilized in the clinical treatment of autoimmune diseases such as rheumatoid arthritis (Luo et al., 2017; Huang et al., 2019a), primary Sjogren’s syndrome (Feng et al., 2019), and ankylosing spondylitis (Huang et al., 2019b). The combination of TGP with conventional therapeutic agents can reduce adverse reactions and have synergistic effects in the treatment of autoimmune diseases (Jiang et al., 2020). In recent years, TGP has also been increasingly used to treat SLE. Previous clinical and experimental studies have shown that TGP can alleviate typical symptoms, increase the expression rate of CD4^+^CD25+T cells, regulate the TLR9/MyD88/NF-KB signaling pathway, reduce the levels of CD40^+^, sVCAM-1, IL-18, VEGF, and MMP-3, and inhibit the expression of inflammatory factors, playing an immunomodulatory and anti-SLE renal damage role. This indicates that TGP may be a potential new therapeutic agent for the modern treatment of SLE (Wu et al., 2020; Wang et al., 2021; Wu et al., 2022).

To date, only one systematic review has evaluated the efficacy of TGP in different courses of treatment for SLE (Chen et al., 2022). However, no study followed the statement of Preferred Reporting Items for Systematic Reviews and Meta-Analyses (PRISMA) to evaluate the efficacy and safety of TGP in combination with different conventional therapeutic agents for the treatment of SLE. This study aims to investigate the efficacy and safety of TGP combined with different conventional therapeutic agents for the treatment of SLE, thus providing an evidence-based basis for future clinical treatment of SLE.

## 2 Methods

### 2.1 Protocol registration

This meta-analysis followed the PRISMA statement (Page et al., 2021). The study protocol was registered at PROSPERO: https://www.crd.york.ac.uk/PROSPERO (Registration number: CRD42021272481).

### 2.2 Search strategy

The method of combining subject words and free words was used to search the randomized controlled trials (RCTs) of TGP for SLE in eight databases including China National Knowledge Infrastructure (CNKI), the Chinese Science Technology Journal Database (VIP), WanFang Database, SinoMed, PubMed, Web of Science, Cochrane Library, and Embase from the inception to March 2022. The detailed search strategies for the eight databases are shown in Supplementary Table S1.

### 2.3 Inclusion criteria

#### 2.3.1 Study types

We included RCTs.

#### 2.3.2 Participant types

The patients were included in accordance with any of the SLE classification criteria (Hochberg, 1997; Petri et al., 2012; Aringer et al., 2019) and were in the active disease stages. There were no restrictions on age and gender.

#### 2.3.3 Intervention types

The control groups were treated according to the European League Against Rheumatism (EULAR) guidelines for SLE (Fanouriakis et al., 2019) or the Chinese treatment guidelines (Chinese Rheumatology Association, 2020) including GC, CTX, HCQ, and TAC, while the experimental groups were treated with TGP combined with the control group drugs.

#### 2.3.4 Outcome types

Primary outcome variables include SLE Disease Activity Index (SLEDAI) and the incidence of adverse reactions.

Secondary outcome variables include complements (C3 and C4), immunoglobulins (IgA, IgG, and IgM), erythrocyte sedimentation rate (ESR), C-reactive protein (CRP), 24 h urine protein, average daily dosage of GC, cumulative dosage of CTX, and recurrence rate.

The efficacy outcomes include: SLEDAI, C3, C4, IgA, IgG, IgM, ESR, CRP, 24 h urine protein, average daily dosage of GC, cumulative dosage of CTX, and recurrence rate. The safety outcomes include the incidence of adverse reactions.

### 2.4 Exclusion criteria

The exclusion criteria include: 1) patients combined with rheumatic immune diseases other than SLE, 2) experimental or control group taking other herbal medicines, 3) literature with duplicate publications, animal experiments, case reports, reviews of progress, and data errors, and 4) inability to obtain the full text.

### 2.5 Study selection

Two researchers (GXH and LCQ) independently searched the eight databases, imported the articles into EndNote X9, and selected articles according to the inclusion and exclusion criteria after deduplication. Any disagreements between the two researchers were resolved by discussion with a third researcher (LH).

### 2.6 Data extraction

Two authors (WSW and CYM) independently extracted the relevant data according to the predefined criteria. The data included: study designs, year of publication, participant characteristics, diagnostic criteria, methodology, intervention and control approaches, treatment duration, outcome measures, and adverse reactions. Any disagreements between the two researchers were resolved by discussion with a third researcher (LSW).

### 2.7 Risk of bias assessment

Two researchers (GXH and WSW) independently assessed the risk of bias of the included 23 studies by referring to the Cochrane Evaluation Manual (5.1.0) (Sterne et al., 2019), mainly from the following seven aspects: 1) random sequence generation, 2) allocation concealment, 3) blinding of outcome assessment, 4) blinding of outcome evaluation, 5) incomplete outcome data, 6) selective reporting, and 7) other biases. Any disagreements between the two researchers were resolved by discussion with the third researcher (LSW).

### 2.8 Data analysis and bias assessment

RevMan 5.3 software was applied to perform data analysis on the continuous and dichotomous data extracted from 23 studies. The relative risk (RR) was used to represent the binary variables, such as the incidence of adverse reactions and the recurrence rate. The standardized mean difference (SMD) was used to represent the continuous variables, such as SLEDAI, C3, C4, IgA, IgG, IgM, ESR, CRP, 24 h urine protein, average daily dosage of GC, and accumulation dosage of CTX. Subgroup analyses were performed according to conventional therapeutic agents. All data were described with the effect size and 95% confidence intervals (CI). When there was significant heterogeneity (I^2^ ≥ 50%, *p*≤ 0.05), a random-effects model was used; otherwise, a fixed-effects model was used. If the heterogeneity is large, sensitivity analyses were carried out by removing the articles one by one and analyzing the causes of heterogeneity by rereading the full text. A funnel plot of adverse reaction rates was plotted to assess publication bias.

### 2.9 Certainty assessment

Two researchers (LSW and GHT) independently assessed the grade of evidence according to the Grading of Recommendations Assessment, Development, and Evaluation (GRADE) methodology, which can be downgraded from five factors (study limitation, consistency of effect, imprecision, indirectness, and publication bias) or upgraded from three reasons (large magnitude of effect, reasonable residual confounding effects, and dose-response gradient). The certainty of evidence was rated as “very low,” “low,” “moderate,” or “high” (Guyatt et al., 2008; Balshem et al., 2011).

## 3 Results

### 3.1 Study selection

**FIGURE 2:**
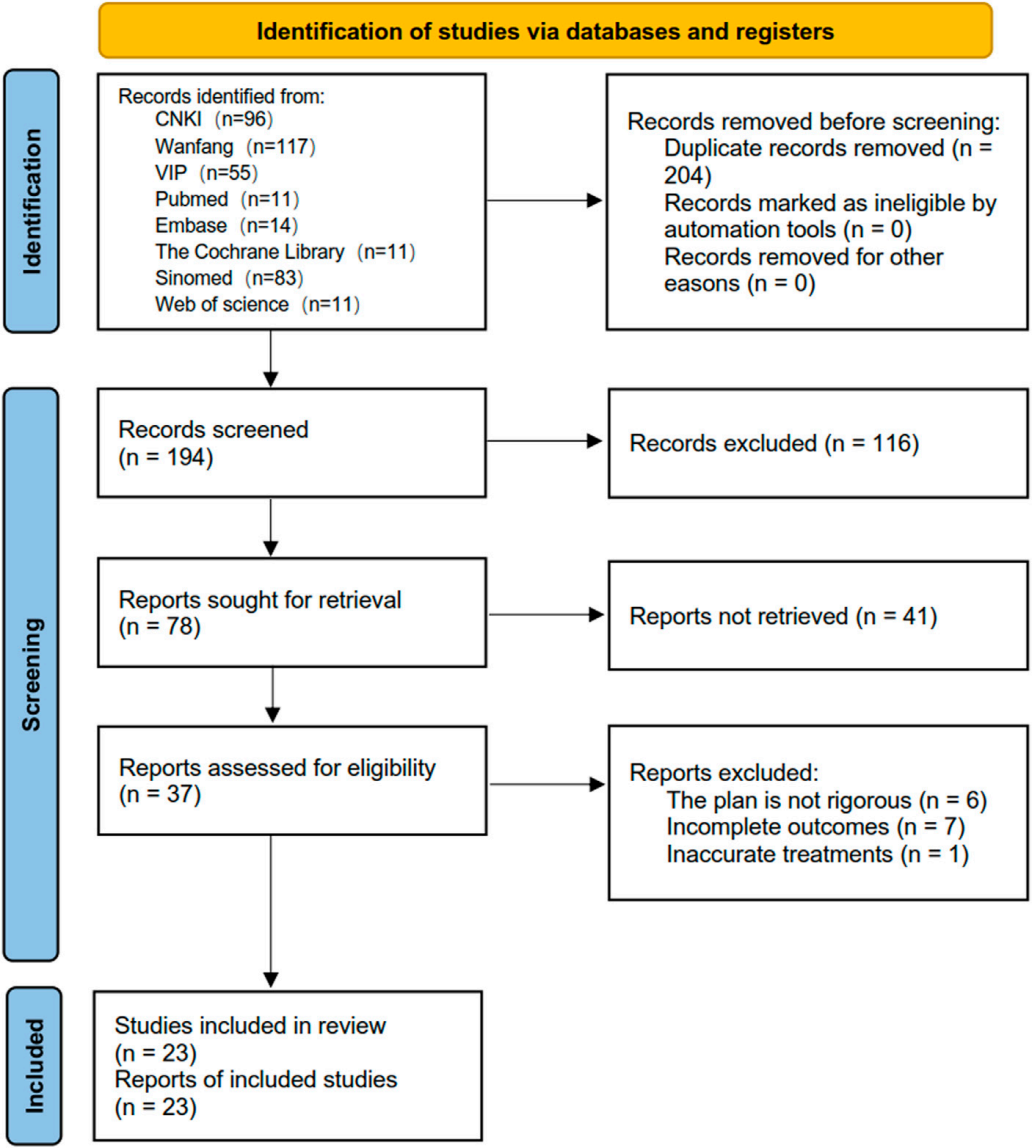
Flow diagram for selection of studies.

A total of 389 articles including 204 duplications were initially retrieved after eight databases were searched by subject words combined with free words. A total of 23 articles were finally included according to the inclusion and exclusion criteria through reading abstracts and full texts. The flow diagram for selection of studies is shown in Figure 2.

### 3.2 Study characteristics

There were 23 RCTs of TGP combined with conventional therapeutic agents for SLE included in this study, including 1,573 patients, with 792 patients overall in the experimental group and 781 patients overall in the control group. All studies were published in Chinese from 2009 to 2020. The sample size ranged from 40 to 106 cases. The control group was treated with conventional therapeutic agents including CTX, TAC, GC, and HCQ, while the experimental group was treated with TGP combined with the drugs of the control groups. The disease course varied from 1 year to 10 years. The duration of treatment ranged from 1 month to 1 year In total 18 studies reported the primary outcomes of SLEDAI and the incidence of adverse reactions. Other studies reported the second outcome. The baseline data were consistent between the two groups. The basic characteristics of the included studies are shown in Table 1 **.**

**TABLE 1:** Basic characteristics of included studies.

Study ID	Gender (male/female)	Sample size (T/C)	Age (years)		Course of disease		Intervention		Treatment duration	Outcome
			T	C	T	C	T	C		
Zhao et al. (2020)	8/98	106 (53/53)	38.14 ± 3.24	34.13 ± 3.22	9.91 ± 2.29y	9.82 ± 2.33y	TGP 0.6 g tid + CTs	GC + HCQ	3 m	①②③⑬
Zhang et al. (2020)	31/33	64 (32/32)	38.2 ± 0.5	37.9 ± 1.2	12.4 ± 0.4 m	11.8 ± 0.7 m	TGP 0.6 g bid + CTs	GC + TAC	6 m	①
Yu et al. (2019)	11/49	60 (30/30)	37.84 ± 5.9 3	37.94 ± 5.82	5.92 ± 1.84y	5.87 ± 1.90y	TGP 0.6 g bid + CTs	GC + CTX	6 m	①②⑦⑧⑩⑪⑬
Yang and Li, (2019)	8/98	106 (53/53)	41.5 ± 4.1	42.7 ± 5.2	3.4 ± 1.1y	3.7 ± 1.3y	TGP 0.6 g tid + CTs	GC	3 m	①②③⑦⑬
Xue and Lyu (2019)	6/54	60 (30/30)	38.15 ± 3.20	38.12 ± 3.25	1.81 ± 0.32y	1.82 ± 0.35y	TGP 0.6 g bid/tid + CTs	GC + HCQ	6 m	②③⑦⑬
Xu, (2020)	21/51	72 (36/36)	36.82 ± 6.29		5.39 ± 1.67y	5.25 ± 1.72y	TGP 0.6 g bid/tid + CTs	GC + CTX	12 m	①⑨⑩⑪⑫⑬
Xu, (2015)	19/34	53 (27/26)	42.7 ± 5.30		5.9 ± 1.7y	5.3 ± 1.1y	TGP 0.6 g bid/tid + CTs	GC + CTX	1 m	①⑨⑩⑪⑬
Wu et al. (2020)	19/55	54 (27/27)	36.82 ± 6.31		3.24 ± 1.20y	3.19 ± 1.02y	TGP 0.6 g bid/tid + CTs	GC + CTX	3 m	②③④⑤⑥⑫⑬
Wang and Wang, (2015)	6/36	42 (21/21)	27.34 ± 7.65	29.28 ± 8.95	NR		TGP 0.6 g tid + CTs	GC	3 m	①⑬
Liu, (2016)	12/74	86 (43/43)	44.2 ± 9.4	44.2 ± 9.4	6.9 ± 3.4y		TGP 0.6 g tid + CTs	GC	NR	④⑤⑫⑬
Li et al. (2018a)	16/74	90 (45/45)	32.15 ± 5.37	33.21 ± 4.94	32.81 ± 9.53 m	32.07 ± 9.86 m	TGP 0.6 g bid + CTs	GC + TAC	6 m	①②③④⑤⑥⑩⑫⑬
Chen, (2013)	4/37	41 (21/20)	27.5 ± 6.7		NR		TGP 0.6 g tid + CTs	GC + CTX	1.5 m	①⑨⑩⑪⑬
Li et al. (2013)	6/88	94 (47/47)	43.1	42.8	7.9y	7.4y	TGP 0.6 g tid + CTs	GC	NR	④⑤⑫
Li and Zheng, (2020)	14/52	66 (33/33)	47.21 ± 7.42		NR		TGP 0.6 g bid + CTs	GC + CTX	1 m	①⑨⑩⑪⑫⑬
Lin and Liu, (2016)	17/34	51 (26/25)	17-34		NR		TGP 0.6 g tid + CTs	GC + CTX	NR	①⑨⑩⑪⑫⑬
Cai et al. (2017)	9/51	60 (30/30)	33.2 ± 5.1		2.47 ± 0.78y	2.56 ± 0.82y	TGP 0.6 g tid + CTs	GC + CTX	6 m	①②⑦⑨⑩⑪⑬
Peng, (2018)	10/30	40 (20/20)	43.3 ± 4.9		5.5 ± 1.2y	5.7 ± 1.1y	TGP 0.6 g tid + CTs	GC + CTX	3 m	①⑨⑩⑪⑫⑬
Xiang, (2020)	17/53	70 (35/35)	39.2 ± 4.3		6.8 ± 1.7y	6.7 ± 1.6y	TGP 0.6 g bid/tid + CTs	GC + CTX	6 m	①⑨⑩⑪
Feng et al. (2017)	19/34	53 (27/26)	42.7 ± 5.30		5.9 ± 1.7y	5.3 ± 1.1y	TGP 0.6 g tid + CTs	GC + CTX	NR	①②⑨⑩⑪⑫
Sun, (2013)	44/52	96 (48/48)	34.6 ± 4.10		2.4 ± 0.8y	2.5 ± 0.7y	TGP 0.6 g tid + CTs	GC + CTX	3 m	①②⑤⑨
Yang, (2016)	11/39	50 (26/24)	43.1 ± 7.2		5.1 ± 1.9y	4.2 ± 2.1y	TGP 0.6 g bid/tid + CTs	GC + CTX	NR	①⑬
Zhu and Wei, (2009)	7/58	65 (35/30)	32 ± 6.5	32 ± 6.5	5m-6y		TGP 0.6 g tid + CTs	GC	3 m	①②③⑦⑧⑩
Wang et al. (2013)	6/88	94 (47/47)	43.1	42.8	7.9y	7.4y	TGP 0.6 g tid + CTs	GC	NR	④⑤⑫⑬

T, test group; C, control group; CTs, control treatments; m, month; y, year; bid, twice a day; tid, three times a day; NR, not reported; GC, glucocorticoids; CTX, cyclophosphamide; TGP, total glucosides of paeony; HCQ, hydroxychloroquine; TAC, tacrolimus; SLEDAI, systemic lupus erythematosus disease activity index; C3, complement 3; C4, complement 4; Ig, Immunoglobulin; ESR, erythrocyte sedimentation rate; CRP, C-reactive protein; ① SLEDAI; ② C3; ③ C4; ④ IgA; ⑤ IgG; ⑥ IgM; ⑦ ESR; ⑧ CRP; ⑨ 24 h urine protein; ⑩ GC, average daily dosage; ⑪ cumulative dosage of CTX; ⑫ recurrence rate; ⑬ incidence of adverse reactions.

### 3.3 Risk of bias assessment

**FIGURE 3:**
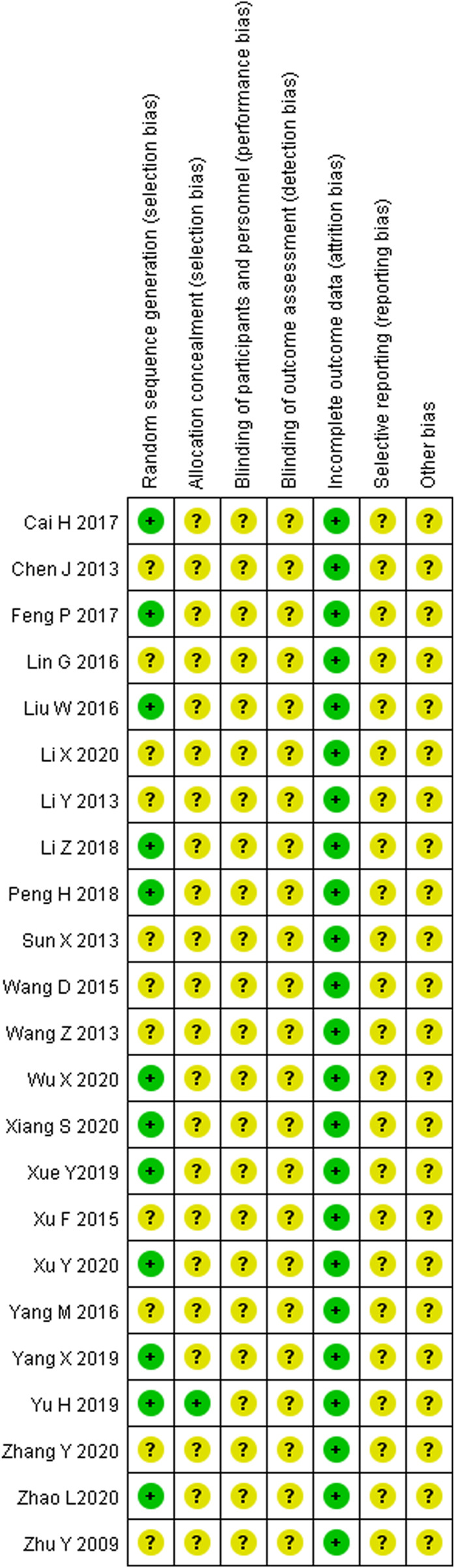
Risk of bias of included studies.

The risk of bias of 23 studies was evaluated according to the Cochrane Evaluation Manual (5.1.0). Seven studies (Lin and Liu, 2016; Cai et al., 2017; Li Z. et al., 2018; Yang and Li, 2019; Wu et al., 2020; Xu, 2020; Zhao et al., 2020) used the random number table method to generate random sequences. Two studies (Peng, 2018; Yu et al., 2019) used the order of admission. One study (Xue and Lyu, 2019) used the coin toss method. One study (Xiang, 2020) used the touch-ball method. The rest of the studies only mentioned randomness and did not elaborate on the method of random sequence generation. None of the studies stated whether a blinding was used, whether allocation concealment was used, or whether the outcome assessment was blinded. All studies were fully reported according to pre-specified outcome measures. The risk of bias for the included studies is shown in Figure 3.

### 3.4 Efficacy outcomes

#### 3.4.1 SLEDAI

**FIGURE 4:**
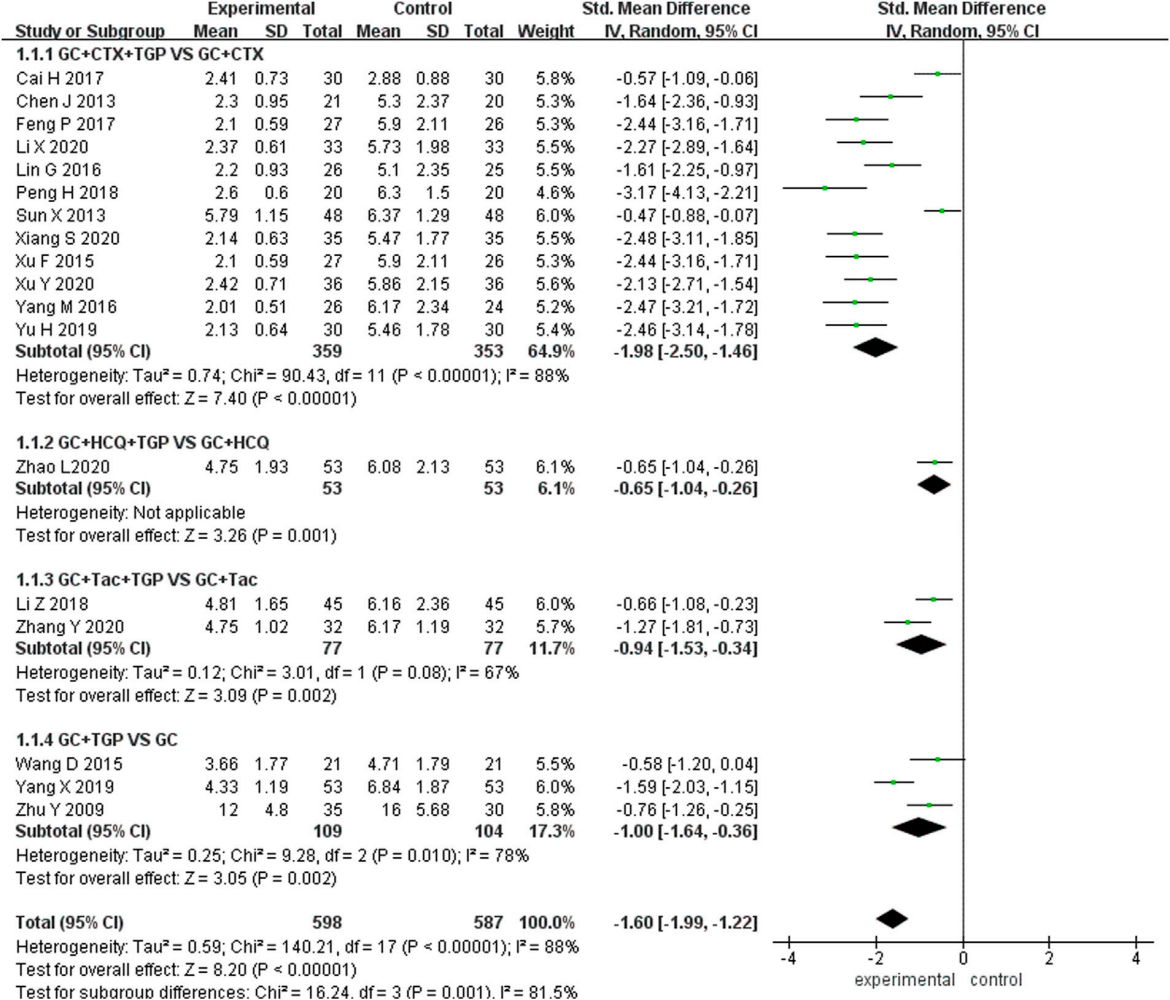
Forest plot of SLEDAI.

A total of 18 studies reported SLEDAI as the primary outcome, including 598 patients overall in the experimental group and 587 patients overall in the control group. Subgroup analysis was performed according to different treatment drugs. Twelve studies (Chen, 2013; Sun, 2013; Xu, 2015; Lin and Liu, 2016; Yang, 2016; Cai et al., 2017; Feng et al., 2017; Peng, 2018; Yu et al., 2019; Li and Zheng, 2020; Xiang, 2020; Xu, 2020) used TGP in combination with GC and CTX. One study (Zhao et al., 2020) used TGP in combination with GC and HCQ. Two studies (Li Z. et al., 2018; Zhang, 2020) used TGP in combination with GC and TAC. Three studies (Zhu and Wei, 2009; Wang and Wang, 2015; Yang and Li, 2019) used TGP in combination with GC. A random-effects model was applied for analysis because of the existence of heterogeneity (I^2^ = 88%, *p* <0.001). Subgroup analysis showed that the SLEDAI score of the experimental group was significantly lower than that of the control group (SMD = −1.98, 95% Cl = [−2.50, −1.46], *p* <0.001; SMD = −0.65, 95% Cl = [−1.04, −0.26], *p* <0.01; SMD = −0.94, 95% Cl = [−1.53, −0.34], *p* <0.01; SMD = −1.00, 95% Cl = [−1.64, −0.36], *p* <0.01). The difference was statistically significant (Figure 4).

Sensitivity analysis was performed to explore the source of heterogeneity by excluding articles sequentially and reading the full text. It was found that two studies (Sun, 2013; Cai et al., 2017) had a significant influence on the result when TGP was combined with GC and CTX treatment. The heterogeneity was reduced after excluding the two articles, (I^2^ = 25% and *p* = 0.21). We merged the data of other studies to analyze (SMD = -2.26, 95% Cl = [-2.52, -2.01], *p* <0.001). The shorter mean duration of disease in patients included in one study (Cai et al., 2017) and the shorter mean disease duration of patients included in another study (Sun, 2013) with more comorbidities and higher disease activity before treatment may be the main reasons for the heterogeneity.

One study (Yang and Li, 2019) had a significant influence on the result when TGP was combined with GC treatment. The heterogeneity was reduced after excluding this study (I^2^ = 0%, *p* = 0.66). Data from other studies was merged for analysis (SMD = −0.69, 95% Cl = [−1.08, −0.29], *p* <0.001). The older average age of patients in this study may be the main reason for the heterogeneity (Supplementary Figure S1).

#### 3.4.2 C3

**FIGURE 5:**
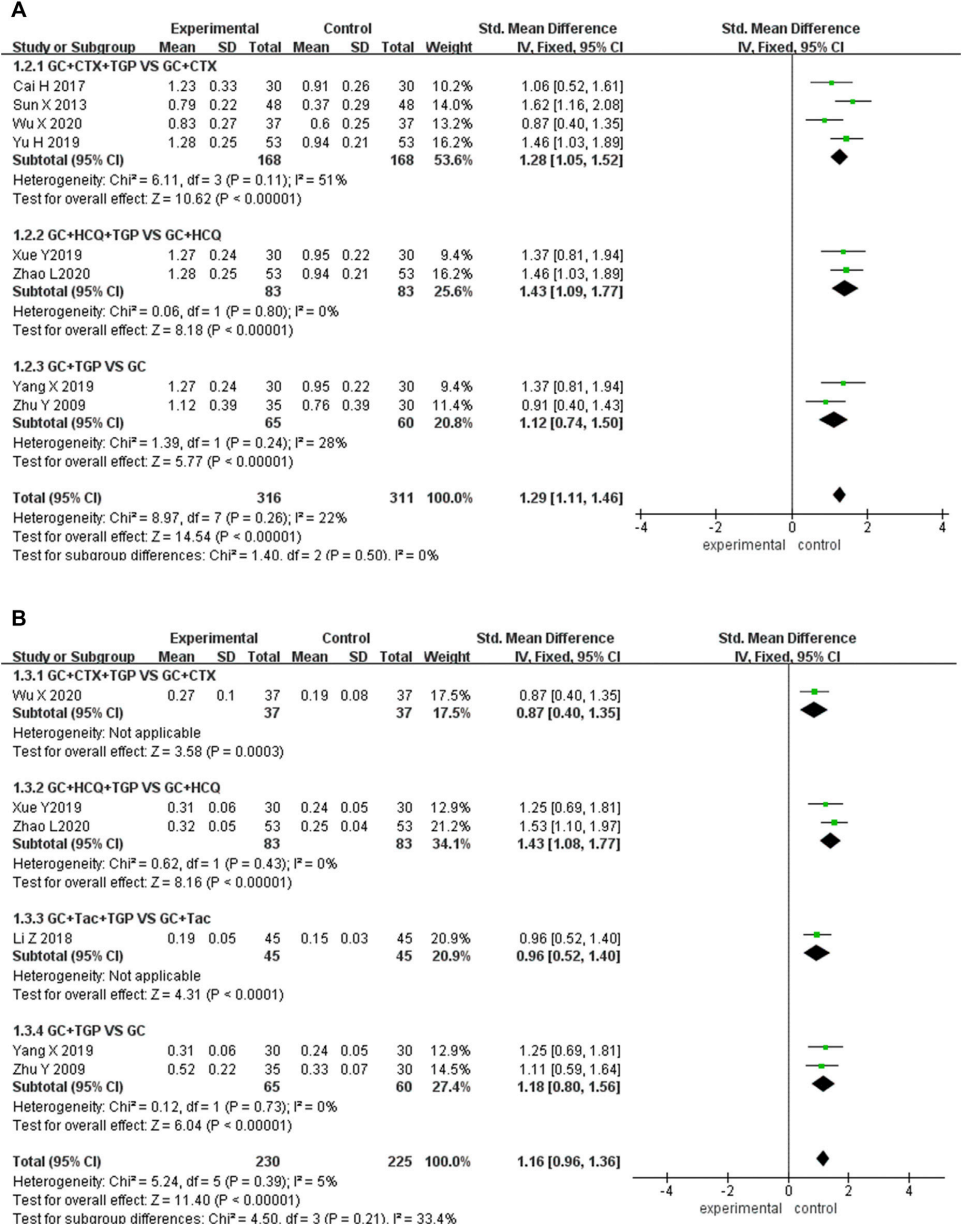
Forest plot of C3 **(A)**, C4 **(B)**.

Eight studies reported C3 as the outcome, including 316 patients overall in the experimental group and 311 patients overall in the control group. Subgroup analysis was performed according to different treatment drugs. Four studies (Sun, 2013; Cai et al., 2017; Yu et al., 2019; Wu et al., 2020) used TGP in combination with GC and CTX. Two studies (Xue and Lyu, 2019; Zhao et al., 2020) used TGP in combination with GC and HCQ. Two studies (Zhu and Wei, 2009; Yang and Li, 2019) used TGP in combination with GC. A fixed-effects model was applied for analysis due to low heterogeneity (I^2^ = 22%, *p* = 0.26). Subgroup analysis showed that C3 in the experimental group was significantly higher than that in the control group (SMD = 1.28, 95% Cl = [1.05, 1.52], *p* <0.001; SMD = 1.43, 95% Cl = [1.09, 1.77], *p* <0.001; SMD = 1.12, 95% Cl = [ 0.74, 1.50], *p* <0.001). The difference was statistically significant (Figure 5A).

#### 3.4.3 C4

Six studies reported C4 as the outcome, including 230 patients overall in the experimental group and 225 patients overall in the control group. Subgroup analysis was performed according to different treatment drugs. One study (Wu et al., 2020) used TGP in combination with GC and CTX. Two studies (Xue and Lyu, 2019; Zhao et al., 2020) used TGP in combination with GC and HCQ. One study (Li Z. et al., 2018) used TGP in combination with GC and TAC. Two studies (Zhu and Wei, 2009; Yang and Li, 2019) used TGP in combination with GC therapy. A fixed-effects model was applied for analysis because of low heterogeneity (I^2^ = 5%, *p* = 0.39). Subgroup analysis showed that C4 in the experimental group was significantly higher than that in the control group (SMD = 0.87, 95% Cl = [0.40, 1.35], *p* <0.001; SMD = 1.43, 95% Cl = [1.08, 1.77], *p* <0.001; SMD = 0.96, 95% Cl = [0.52, 1.40], *p* <0.001; SMD = 1.18, 95% Cl = [0.80, 1.56], *p* <0.001). The difference was statistically significant (Figure 5B).

#### 3.4.4 IgA

**FIGURE 6:**
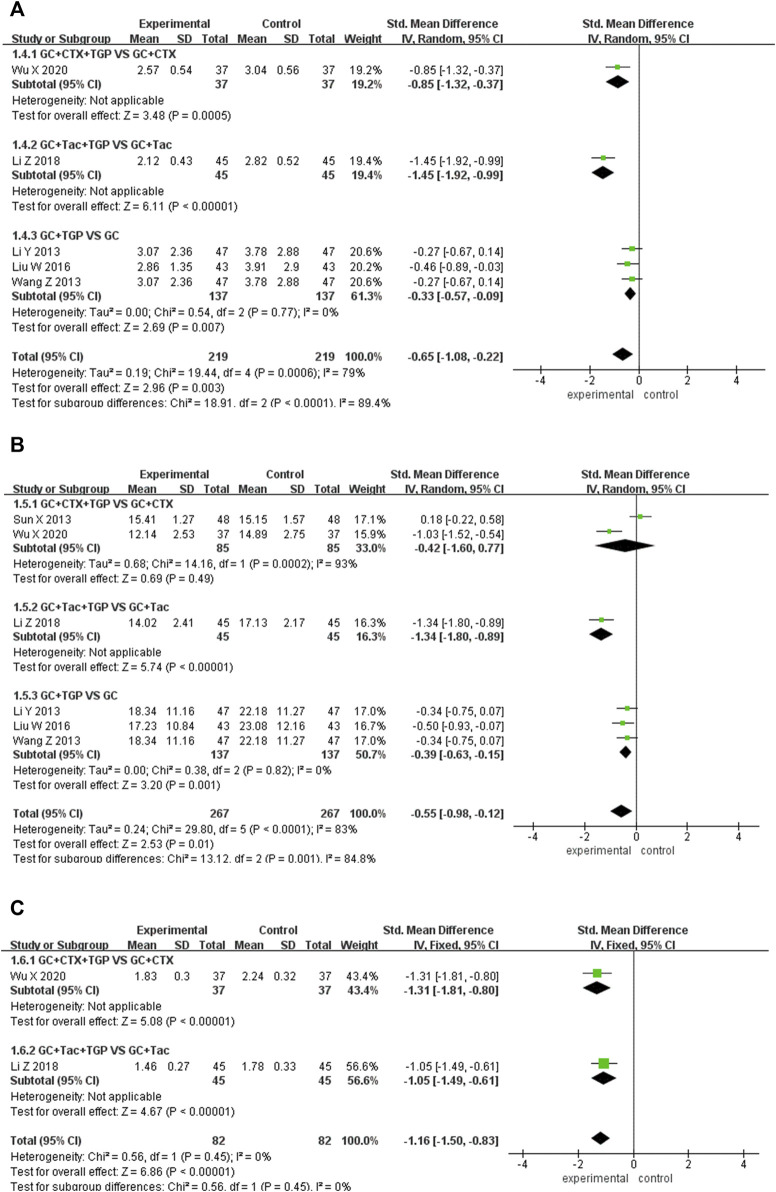
Forest plot of IgA **(A)**, IgG **(B)**, IgM **(C)**.

Five studies reported IgA as the outcome, including 215 patients overall in the experimental group and 191 patients overall in the control group. Subgroup analysis was performed according to different treatment drugs. One study (Wu et al., 2020) used TGP in combination with GC and CTX. One study (Li Z. et al., 2018) used TGP in combination with GC and HCQ. Three studies (Li, 2013; Liu, 2016; Peng, 2018) used TGP in combination with GC. A random-effects model was applied for analysis because of the existence of heterogeneity (I^2^ = 79%, *p* <0.001). Subgroup analysis showed that IgA in the experimental group was lower than that in the control group (SMD = -0.85, 95% Cl = [−1.32, −0.37], *p* <0.001; SMD = −1.45, 95% Cl = [−1.92, −0.99], *p* <0.001; SMD = −0.33, 95% Cl = [ −0.57, −0.09], *p* <0.01). The difference was statistically significant (Figure 6A).

Sensitivity analysis was performed to explore the source of heterogeneity by excluding articles sequentially and reading the full text. Two studies (Li Z. et al., 2018; Wu et al., 2020) were found to have a significant impact on the result. The heterogeneity was reduced after excluding the two articles (I^2^ = 0%, *p* = 0.77). We merged the data of other studies for analysis (SMD = −0.33, 95% Cl = [−0.57, −0.09], *p* < 0.01). It was found that TGP in combination with different drug treatments may be responsible for the heterogeneity (Supplementary Figure S2).

#### 3.4.5 IgG

Six studies reported IgG as the outcome, including 267 patients overall in the experimental group and 267 patients overall in the control group. Subgroup analysis was performed according to different treatment drugs. Two studies (Sun, 2013; Wu et al., 2020) used TGP in combination with GC and CTX. One study (Li Z. et al., 2018) used TGP in combination with GC and TAC. Three studies (Li, 2013; Wang et al., 2013; Liu, 2016) used TGP in combination with GC. A random-effects model was applied for analysis because of the existence of heterogeneity (I^2^ = 83%, *p* <0.001). Subgroup analysis showed that the IgG in the experimental group was lower than that in the control group (SMD = -0.42, 95% Cl = [−1.60, 0.77], *p* < 0.001; SMD = −1.34, 95% Cl = [−1.80, −0.89], *p* < 0.001; SMD = −0.39, 95% Cl = [−0.63, −0.15], *p* < 0.01). The difference was statistically significant (Figure 6B).

Sensitivity analysis was performed to explore the source of heterogeneity by excluding articles sequentially and reading the full text. Three studies (Sun, 2013; Li Z. et al., 2018; Wu et al., 2020) were found to have a significant impact on the result. The heterogeneity was reduced after excluding the three articles (I^2^ = 0%, *p* = 0.82). We merged the data of other studies to analyze (SMD = −0.39, 95% Cl = [−0.63, −0.15], *p* < 0.01). It was found that TGP in combination with different drug treatments may be responsible for heterogeneity (Supplementary Figure S3).

#### 3.4.6 IgM

Two studies reported IgM as the outcome, including 82 patients overall in the experimental group and 82 patients overall in the control group. Subgroup analysis was performed according to different treatment drugs. One study (Wu et al., 2020) used TGP in combination with GC and CTX. One study (Li Z. et al., 2018) used TGP in combination with GC and TAC. A fixed-effects model was applied for analysis because of low heterogeneity (I^2^ = 0%, *p* = 0.45). Subgroup analysis showed that the IgM in the experimental group was lower than that in the control group (SMD = −1.31, 95% Cl = [−1.81, −0.80], *p* < 0.001; SMD = −1.05, 95% Cl = [−1.49, −0.61], *p* < 0.001). The difference was statistically significant (Figure 6C).

#### 3.4.7 ESR

**FIGURE 7:**
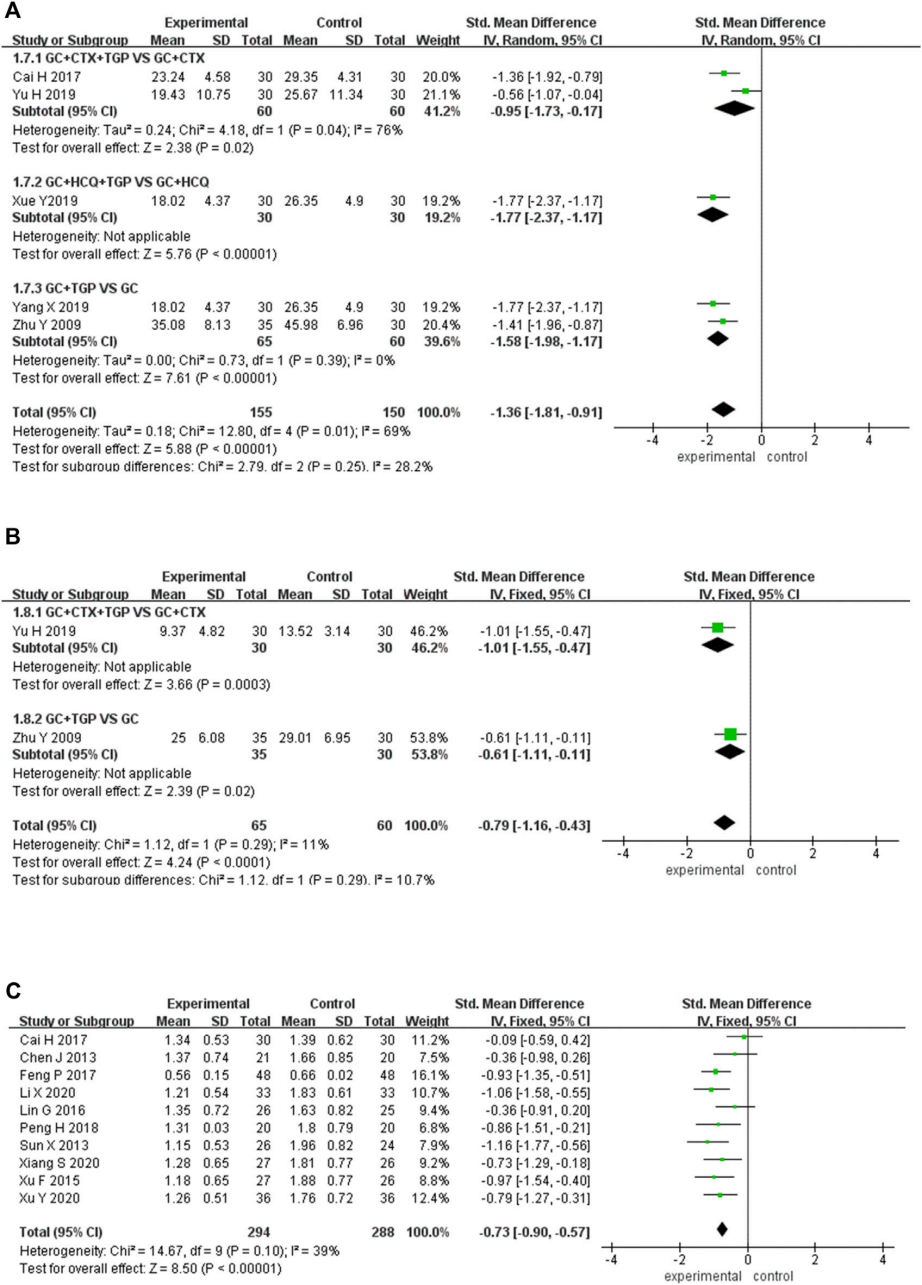
Forest plot of ESR **(A)**, CRP **(B)**, 24h urine protein **(C)**.

Five studies reported ESR as the outcome, including 155 patients overall in the experimental group and 150 patients overall in the control group. Subgroup analysis was performed according to different treatment drugs. Two studies (Cai et al., 2017; Yu et al., 2019) used TGP in combination with GC and CTX. One study (Xue and Lyu, 2019) used TGP in combination with GC and HCQ. Two studies (Zhu and Wei, 2009; Yang and Li, 2019) used TGP in combination with GC. A random-effects model was applied for analysis because of the existence of heterogeneity (I^2^ = 69%, *p* < 0.01). Subgroup analysis showed that the ESR of the experimental group was lower than that in the control group (SMD = −0.95, 95% Cl = [−1.73, −0.17], *p* <0.05; SMD = −1.77, 95% Cl = [−2.37, −1.17], *p* <0.001; SMD = −1.58, 95% Cl = [−1.98, −0.17], *p* <0.001). The difference was statistically significant (Figure 7A).

Sensitivity analysis was performed to explore the source of heterogeneity by excluding articles sequentially and reading the full text. It was found that one study (Yu et al., 2019) had an important impact on the result. The heterogeneity was reduced after excluding this article (I^2^ = 0%, *p* = 0.63). We merged the data of other studies to analyze (SMD = −1.56, 95% Cl = [−1.85, −1.27], *p* < 0.001). The imprecise result of the study may be the main reason for the heterogeneity. (Supplementary Figure S4).

#### 3.4.8 CRP

Two studies reported CRP as the outcome, including 65 patients overall in the experimental group and 60 patients overall in the control group. Subgroup analysis was performed according to different treatment drugs. One study (Yu et al., 2019) used TGP in combination with GC and CTX. One study (Zhu and Wei, 2009) used TGP in combination with GC. A fixed-effects model was applied for analysis because of low heterogeneity (I^2^ = 11%, *p* = 0.29). Subgroup analysis showed that the CRP of the experimental group was lower than that of the control group (SMD = −1.01, 95% Cl = [−1.55, −0.47], *p* <0.001; SMD = −0.61, 95% Cl = [−1.11, -0.11], *p* < 0.05). The difference was statistically significant (Figure 7B).

#### 3.4.9 24 h urine protein

Ten studies reported 24 h urine protein as the outcome, including 294 patients overall in the experimental group and 288 patients overall in the control group. All the ten studies (Chen, 2013; Sun, 2013; Xu, 2015; Lin and Liu, 2016; Cai et al., 2017; Feng et al., 2017; Peng, 2018; Li and Zheng, 2020; Xiang, 2020; Xu, 2020) used TGP in combination with GC and CTX. A fixed-effects model was applied for analysis because of low heterogeneity (I^2^ = 39%, *p* = 0.10). Subgroup analysis showed that the 24 h urine protein in the experimental group was significantly lower than that in the control group (SMD = −0.73, 95% Cl = [−0.90, −0.57], *p* < 0.001). The difference was statistically significant (Figure 7C).

#### 3.4.10 Average daily dosage of GC

**FIGURE 8:**
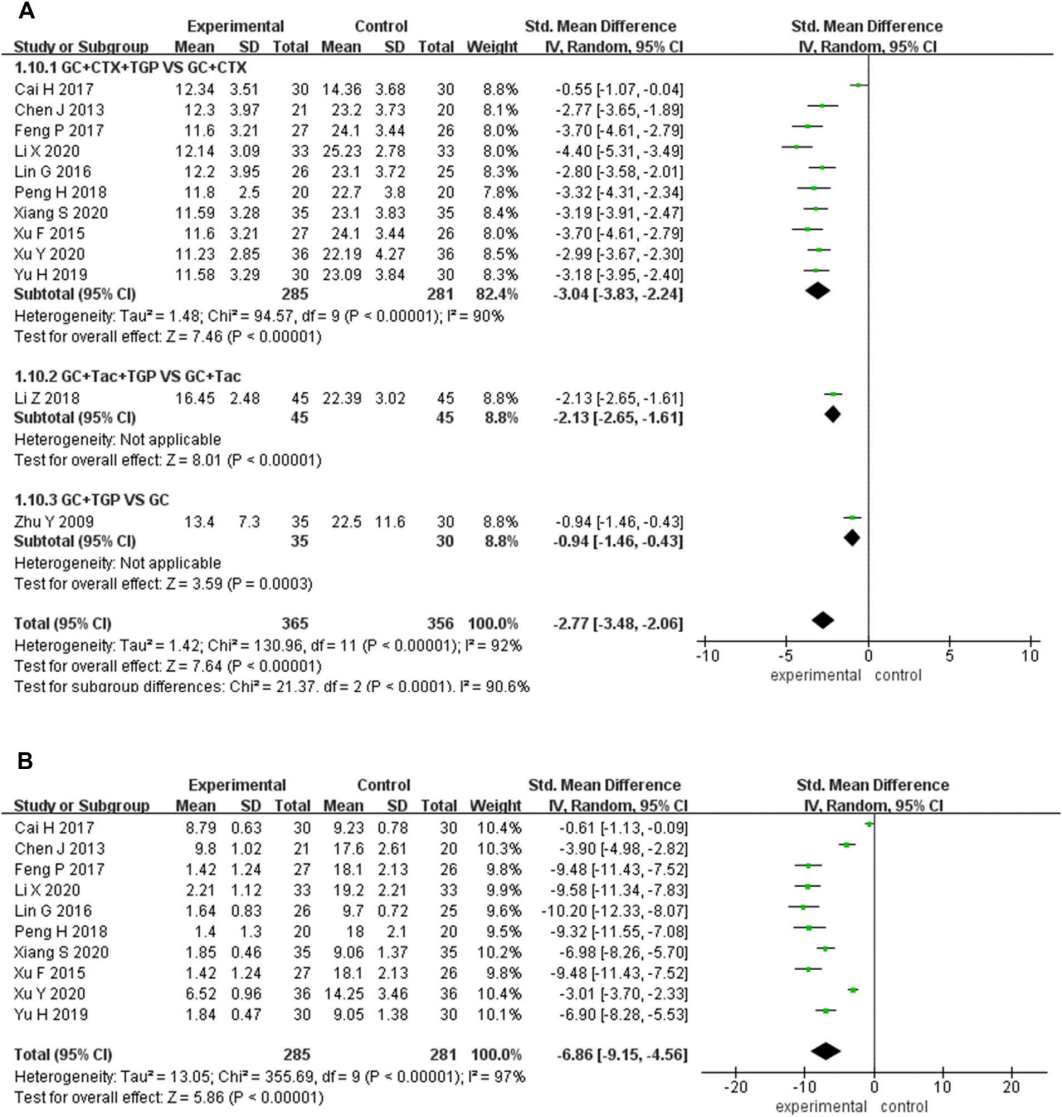
Forest plot of average daily dosage of GC **(A)**, cumulative dosage of CTX **(B)**.

Twelve studies reported the average daily dosage of GC as the outcome, including 365 patients overall in the experimental group and 356 patients overall in the control group. Subgroup analysis was performed according to different treatment drugs. Ten studies (Chen, 2013; Xu, 2015; Lin and Liu, 2016; Cai et al., 2017; Feng et al., 2017; Peng, 2018; Yu et al., 2019; Li and Zheng, 2020; Xiang, 2020; Xu, 2020) used TGP in combination with GC and CTX. One study (Li Z. et al., 2018) used TGP in combination with GC and TAC. One study (Zhu and Wei, 2009) used TGP in combination with GC. A random-effects model was applied for analysis because of the existence of heterogeneity (I^2^ = 92%, *p* < 0.001). Subgroup analysis showed that the average daily dosage of GC in the experimental group was lower than that of the control group (SMD = -3.04, 95% Cl = [-3.83, -2.24], *p* <0.001; SMD = −2.13, 95% Cl = [−2.65, −1.61], *p* < 0.001; SMD = −0.94, 95% Cl = [−1.46, −0.43], *p* < 0.001). The difference was statistically significant (Figure 8A).

Sensitivity analysis was performed to explore the source of heterogeneity by excluding articles sequentially and reading the full text. It was found that three studies (Zhu and Wei, 2009; Cai et al., 2017; Li Z. et al., 2018) had a significant impact on the result. The heterogeneity was reduced after excluding the three articles (I^2^ = 28%, *p* = 0.20). We merged the data of other studies to analyze (SMD = −3.30, 95% Cl = [−3.62, −2.97], *p* < 0.001). One study (Cai et al., 2017) had a shorter average course of disease, and the other two studies (Zhu and Wei, 2009; Li Z. et al., 2018) were combined with different drugs, which may be the main reason for heterogeneity. (Supplementary Figure S5).

#### 3.4.11 Cumulative dosage of CTX

Ten studies reported the cumulative dosage of CTX, including 285 patients overall in the experimental group and 281 patients overall in the control group. All the ten studies (Chen, 2013; Xu, 2015; Lin and Liu, 2016; Cai et al., 2017; Feng et al., 2017; Peng, 2018; Yu et al., 2019; Li and Zheng, 2020; Xiang, 2020; Xu, 2020) used TGP in combination with GC and CTX. A random-effects model was applied for analysis because of the existence of heterogeneity (I^2^ = 97%, *p* < 0.001). Subgroup analysis showed that the cumulative dosage of CTX in the experimental group was significantly lower than that of the control group (SMD = −6.86, 95% Cl = [−9.15, −4.56], *p* < 0.001). The difference was statistically significant (Figure 8B).

Sensitivity analysis was performed to explore the source of heterogeneity by excluding articles sequentially and reading the full text. It was found that five studies (Chen, 2013; Cai et al., 2017; Yu et al., 2019; Xiang, 2020; Xu, 2020) had a significant impact on the result. The heterogeneity was reduced after excluding the five studies (I^2^ = 0%, *p* = 0.98). We merged the data of other studies to analyze (SMD = −9.60, 95% Cl = [−10.49, −8.72], *p* < 0.001). The large differences in the mean age and treatment duration of the patients in the five studies compared with the other studies may account for the heterogeneity (Supplementary Figure S6).

#### 3.4.12 Recurrence rate

**FIGURE 9:**
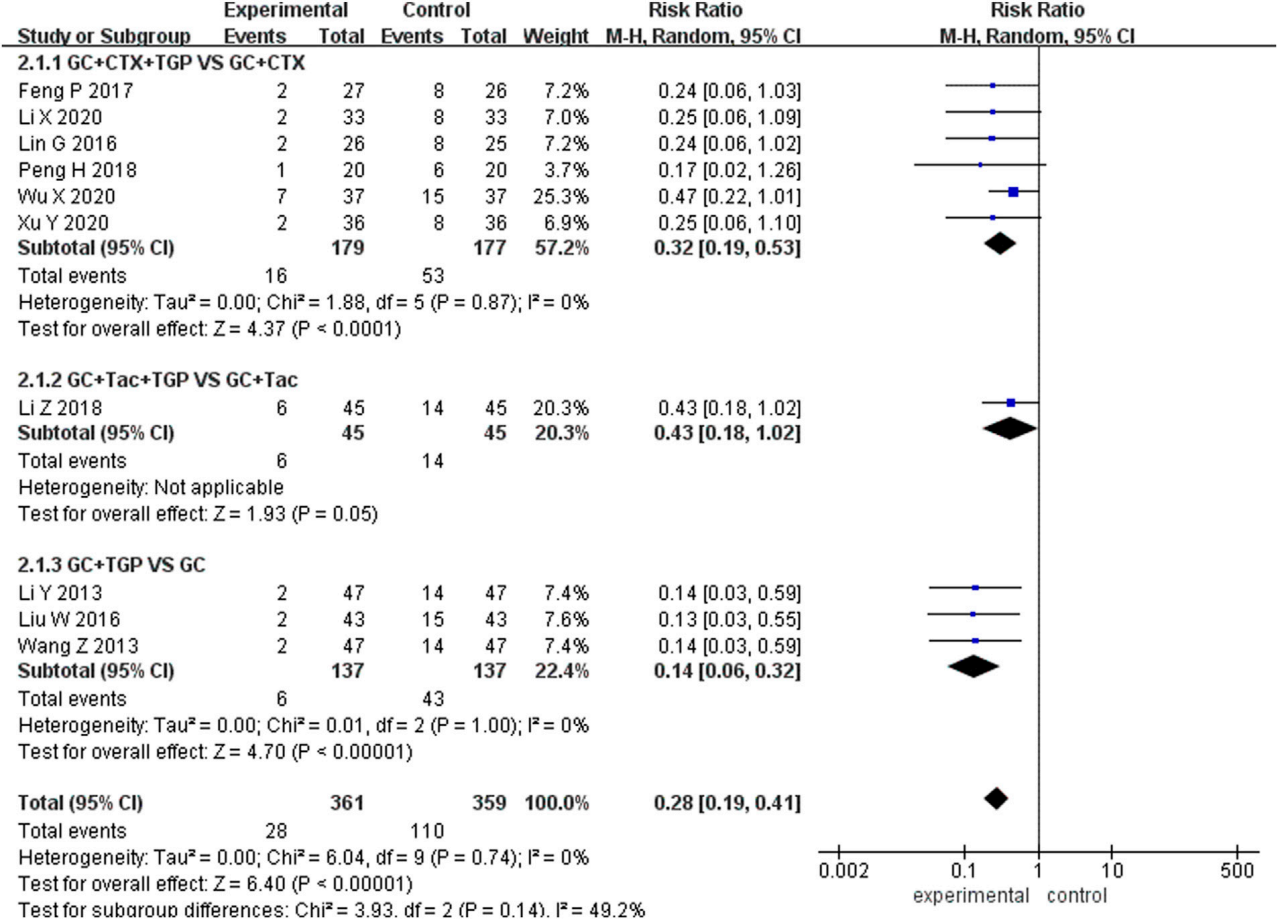
Forest plot of recurrence rate.

Ten studies reported the recurrence rate, including 361 patients overall in the experimental group and 359 patients overall in the control group. Subgroup analysis was performed according to different treatment drugs. Six studies (Lin and Liu, 2016; Feng et al., 2017; Peng, 2018; Li and Zheng, 2020; Wu et al., 2020; Xu, 2020) used TGP in combination with GC and CTX. One study (Li Z. et al., 2018) used TGP in combination with GC and TAC. Three studies (Li, 2013; Wang et al., 2013; Liu, 2016) used TGP in combination with GC. A fixed-effects model was applied for analysis because of low heterogeneity (I^2^ = 0%, *p* = 0.74). Subgroup analysis showed that the recurrence rate of the experimental group was lower than that of the control group (RR = 0.32, 95% Cl = [0.19, 0.53], *p* < 0.001; RR = 0.43, 95% Cl = [0.18, 1.02], *p* < 0.05; RR = 0.14, 95% Cl = [0.06, 0.32], *p* < 0.001). The difference was statistically significant (Figure 9).

### 3.5 Safety outcomes

#### 3.5.1 Incidence of adverse reactions

**FIGURE 10:**
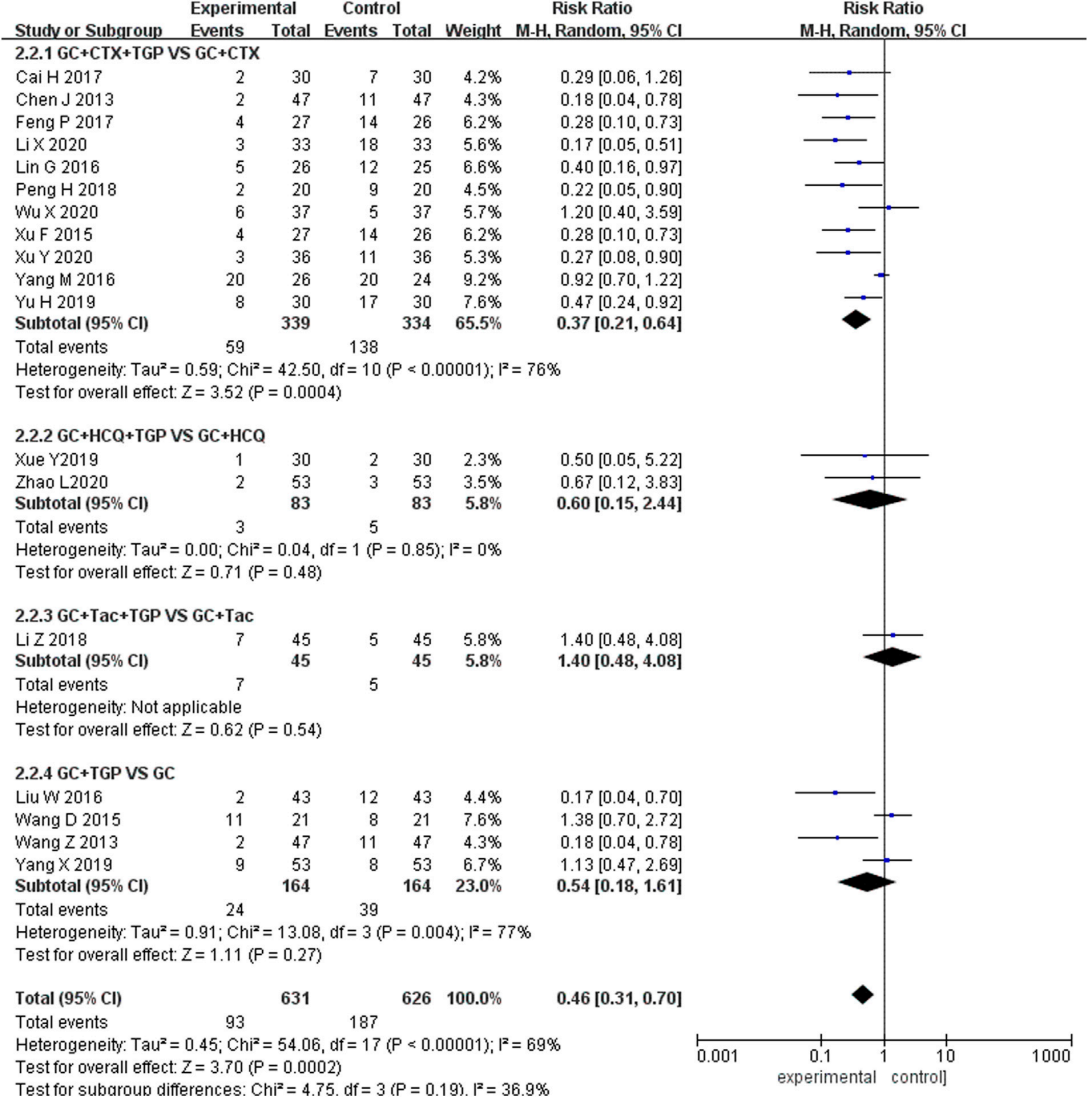
Forest plot of adverse reactions.

Eighteen studies reported the incidence of adverse reactions, including 631 patients overall in the experimental group and 626 patients overall in the control group. Subgroup analysis was performed according to the different treatment drugs. Eleven studies (Chen, 2013; Xu, 2015; Lin and Liu, 2016; Yang, 2016; Cai et al., 2017; Feng et al., 2017; Peng, 2018; Yu et al., 2019; Li and Zheng, 2020; Wu et al., 2020; Xu, 2020) used TGP in combination with GC and CTX. Two studies (Xue and Lyu, 2019; Zhao et al., 2020) used TGP in combination with GC and HCQ. One study (Li Z. et al., 2018) used TGP in combination with GC and TAC. Four studies (Wang et al., 2013; Wang and Wang, 2015; Liu, 2016; Yang and Li, 2019) used TGP in combination with GC. A random-effects model was applied for analysis because of the existence of heterogeneity (I^2^ = 69%, *p* <0.001). Subgroup analysis showed that the incidence of adverse reactions in TGP combined with GC and CTX treatment was lower than that in the control group (RR = 0.37, 95% Cl = [0.21, 0.64], *p* <0.001), and the difference was statistically significant. The remaining three groups had no significant advantage over the control group in the incidence of adverse reactions (RR = 0.60, 95% Cl = [0.15, 2.44], *p* = 0.48; RR = 1.40, 95% Cl = [0.48, 4.08], *p* = 0.54; RR = 0.54, 95% Cl = [0.18, 1.61], *p* = 0.27) (Figure 10).

Sensitivity analysis was performed to explore the source of heterogeneity by excluding articles sequentially and reading the full text. Four studies (Wang and Wang, 2015; Yang, 2016; Yang and Li, 2019; Wu et al., 2020) were found to have a significant impact on the result. The heterogeneity was reduced after excluding the four studies (I^2^ = 3%, *p* = 0.41). We merged the data of other studies to analyze (SMD = 0.34, 95% Cl = [ 0.25, 0.46], *p* < 0.001). The inconsistency in the evaluation criteria for the occurrence of adverse reactions among the four studies may be the main reason for heterogeneity (Supplementary Figure S7).

### 3.6 Publication bias

**FIGURE 11:**
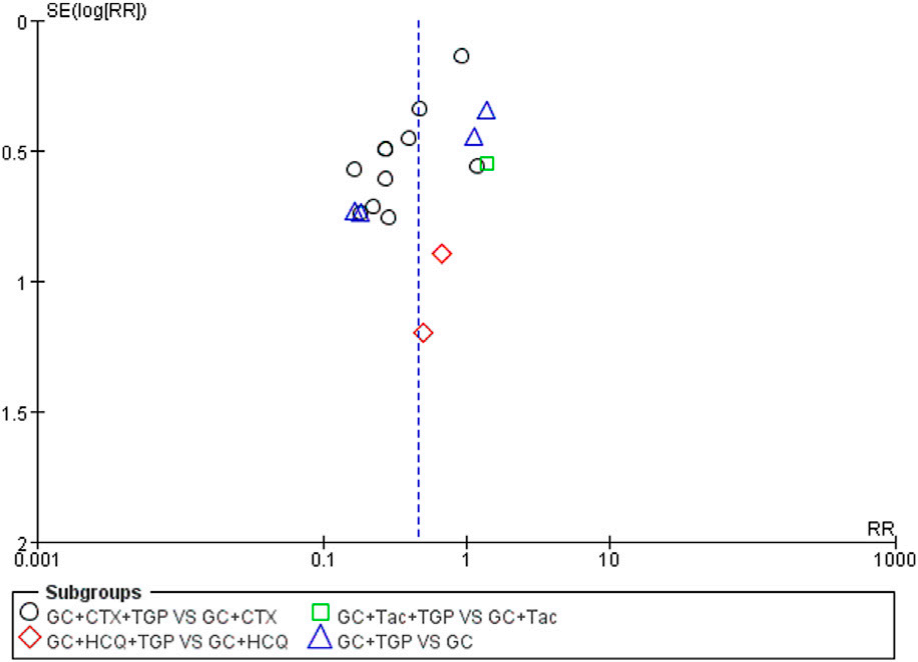
Funnel plot of incidence of adverse reactions.

We used RevMan 5.3 software to draw a funnel plot for the incidence of adverse reactions to analyze publication bias. The results show that the two sides of the funnel plot are asymmetrical, indicating some publication bias in the included literature, which may be related to the evaluation criteria for the incidence of adverse reactions, the small sample size of individual studies, and unpublished negative results (Figure 11).

### 3.7 GRADE assessment

According to the GRADE methodology, the SLEDAI score, the incidence of adverse reactions, and the recurrence rate of TGP combined with different drugs for SLE were evaluated with evidence levels of “medium” and “low.” The results of the GRADE evaluation are shown in Table 2.

**TABLE 2:** GRADE Summary of outcomes of TGP combined with different Western medicines for SLE.

Outcome	Anticipated absolute effect (95% CI)		Relative effect (95% CI)	No. of participants (studies)	Certainty of evidence (GRADE)
	Risk with C	Risk difference with T			
SLEDAI	mean (2.88–6.84)	SMD 1.6 (1.99 and 1.22)	-	1,185 (18 RCTs)	⊕⊕⊕○^1^ < Moderate
SLEDAI (GC + CTX + TGP)	mean (2.88–6.37)	SMD 1.98 (2.5 and 1.46)	-	712 (12 RCTs)	⊕⊕○○^1^ ^,^ ^2^ < Low
SLEDAI (GC + HCQ + TGP)	mean (6.08–6.08)	SMD 0.65 (1.04 and 0.26)	-	106 (1 RCT)	⊕⊕⊕○^1^ < Moderate
SLEDAI (TGP + GC + TAC)	mean (6.16–6.17)	SMD 0.94 (1.53 and 0.34)	-	154 (2 RCTs)	⊕⊕○○^1^ ^,^ ^2^ < Low
SLEDAI (TGP + GC)	mean (4.71–6.84)	SMD 0.69 (1.08 and 0.29)	-	107 (2 RCTs)	⊕⊕⊕○^1^ < Moderate
Incidence of adverse reactions	30 per 100	14 per 100 (9 and 21)	RR = 0.46 (0.31 and 0.70)	1,257 (18 RCTs)	⊕⊕○○^1^ ^,^ ^2^ < Low

Incidence of adverse reactions (TGP + GC + CTX)	41 per 100	13 per 100 (9 and 18)	RR 0.31 (0.22, 0.43)	549 (9 RCTs)	⊕⊕○○^1^ ^,^ ^2^ < Low

Incidence of adverse reactions (TGP + GC + HCQ)	6 per 100	4 per 100 (1 and 15)	RR 0.60 (0.15, and 2.44)	166 (2 RCTs)	⊕⊕⊕○^1^ < Moderate

Incidence of adverse reactions (TGP + GC + TAC)	11 per 100	16 per 100 (5 and 45)	RR 1.40 (0.48, 4.08)	90 (1 RCT)	⊕⊕⊕○^1^ < Moderate

Incidence of adverse reactions (TGP + GC)	26 per 100	4 per 100 (2 and 12)	RR 0.17 (0.06 and 0.48)	180 (2 RCTs)	⊕⊕○○^1^ ^,^ ^2^ < Low

Recurrence rate	31 per 100	9 per 100 (6 and 13)	RR 0.28 (0.19, and 0.41)	359 (10 RCTs)	⊕⊕⊕○^1^ < Moderate

^1^ There is a risk of bias in the implementation of random methods.

^2^ I^2^≥50% with large heterogeneity.

## 4 Discussion

SLE is a chronic autoimmune disease that requires clinical monitoring of multiple indicators to assess disease activity to guide clinical treatment. Among the SLE disease activity assessment tools, SLEDAI is widely adopted by clinicians because of its relatively easy and time-consuming assessment process (Chinese Rheumatology Association, 2020). ESR is a non-specific inflammatory index, but it is a valid indicator for the disease activity assessment in patients with non-infectious SLE (Dima et al., 2016). Low complement is an important serological manifestation of SLE and decreased C3 and C4 can predict SLE flares (Durcan and Petri, 2020). Urine protein reflects renal pathology, and studies have found a positive correlation between 24 h urine protein and SLE disease activity (Li M. et al., 2018). In this study, we evaluated the efficacy and safety of TGP in combination with different conventional therapeutic agents for the treatment of SLE by SLEDAI and these aforementioned indicators provide an evidence-based basis for the future use of TGP in the clinical management of SLE.

Twenty three RCTs involving 1,573 patients were included in this study. The results of the meta-analysis showed that TGP combined with GC and CTX could improve the SLEDAI score, C3, C4, IgA, IgG, IgM, ESR, CRP, 24 h urinary protein, recurrence rate, incidence of adverse reactions, and reduce the average daily dosage of GC and cumulative dosage of CTX. TGP combined with GC and HCQ has more advantages in improving the SLEDAI score, C3, C4, ESR, and 24 h urine protein. TGP combined with GC and TAC improved the SLEDAI score, IgA, IgG, IgM, recurrence rate, and reduced the average daily dosage of GC. TGP combined with GC was more advantageous in improving the SLEDAI score, C3, C4, IgA, IgG, ESR, CRP, 24 h urine protein, recurrence rate, and reduced the average daily dosage of GC. TGP combined with GC and HCQ or GC and TAC or GC had no significant advantage in terms of the incidence of adverse effects compared with the control group. It shows that TGP combined with different conventional therapeutic agents can effectively and safely reduce SLE disease activity. The certainty of the evidence ranges from low to moderate.

This study strictly follows the PRISMA and GRADE methodology and reports a systematic review of the evidence on the efficacy and safety of TGP in combination with different conventional therapeutic agents for the treatment of SLE. However, our review still has certain limitations: 1) many of the included studies did not describe the implementation process of the randomization protocol, and none of them stated whether the allocation-concealed dosing method was used or whether it was blinded, 2) the efficiency, adverse reaction, and recurrence rate were used as outcome observation indicators, but the evaluation criteria were inconsistent, 3) the baseline information of included patients, e.g., age range, cause of disease, extent of SLEDAI, and the duration of treatment had large deviations, 4) the sample size of individual studies was small, 5) potential causal relationships between adverse reactions and TGP were not assessed. In conclusion, the quality of the included studies is relatively low. Therefore, larger and more rigorous RCTs focusing on TGP for the treatment of SLE are needed to verify.

For future studies, we make the following recommendations. First, clinical studies should use enhanced methodological quality, such as proper application of randomization, allocation concealment, and blinding. Second, the design of clinical study protocols is equally important. Investigators should refer to the latest guidelines for controlled trials of SLE treatment. Third, the selection of outcome indicators in clinical studies should clearly specify the criteria for the evaluation of efficacy and adverse effects. Overall, future studies should focus on adopting standardized clinical study designs as a way to improve the methodological and reporting quality of systematic evaluation or meta-analysis, so as to make the conclusion more clinically applicable and provide reliable evidence for clinicians.

## 5 Conclusion

According to the current limited evidence, TGP as an adjuvant therapy, combined with conventional therapeutic agents, may effectively and safely reduce disease activity in SLE patients. Therefore, TGP may become a promising complementary therapy whose long-term efficacy should be explored in the future. However, due to the low quality of both the methods and evidence, we should be cautious about the conclusion drawn from the included studies.

## Data Availability

The original contributions presented in the study are included in the article/Supplementary Material; further inquiries can be directed to the corresponding author.
