# Supplementary material for: Efficacy and safety of total glucosides of paeony in the treatment of systemic lupus erythematosus: A systematic review and meta-analysis
Source: Front Pharmacol. 2022 Dec 7;13:932874. doi: 10.3389/fphar.2022.932874 (PMC9768345; doi:10.3389/fphar.2022.932874)
Supplement: Supplementary file 1 [file Table1.docx]

**Supplementary materials**

**Table S1 Details of the Literature Search Strategy**

| **PubMed** | |
| --- | --- |
| **Number** | **Search terms** |
| #1 | Lupus Erythematosus, Systemic[MeSH Terms] |
| #2 | (Systemic Lupus Erythematosus[Title/Abstract]) or (Lupus Erythematosus Disseminatus[Title/Abstract]) or (Libman-Sacks Disease[Title/Abstract]) or (Disease, Libman-Sacks[Title/Abstract]) or (Libman Sacks Disease[Title/Abstract]) |
| #3 | #1 OR #2 |
| #4 | (paeon∗[Title/Abstract]) or (TGP[Title/Abstract]) |
| #5 | #3 AND #4 |
| **Embase** | |
| **Number** | **Search terms** |
| #1 | exp systemic lupus erythematosus/ |
| #2 | "Systemic Lupus Erythematosus":ti,ab OR "Lupus Erythematosus Disseminatus":ti,ab OR "Libman-Sacks Disease":ti,ab OR "Disease, Libman-Sacks":ti,ab OR "Libman Sacks Disease":ti,ab |
| #3 | #1 or #2 |
| #4 | "paeon∗":ti,ab OR "TGP":ti,ab |
| #5 | #3 and #4 |
| **Cochrane Library** | |
| **Number** | **Search terms** |
| #1 | MeSH descriptor: [Lupus Erythematosus, Systemic] explode all trees |
| #2 | (Systemic Lupus Erythematosus):ti,ab,kw OR (Lupus Erythematosus Disseminatus):ti,ab,kw OR (Libman-Sacks Disease):ti,ab,kw (Disease, Libman-Sacks):ti,ab,kw OR (Libman Sacks Disease):ti,ab,kw |
| #3 | (paeon∗):ti,ab,kw OR (TGP):ti,ab,kw |
| #4 | #1 OR #2 |
| #5 | #3 AND #4 |
| **Web of science** | |
| #1 | TS=(paeon∗ OR TGP) |
| #2 | TS=(Systemic Lupus Erythematosus OR Lupus Erythematosus Disseminatus OR Libman-Sacks Disease OR Disease, Libman-Sacks OR (Libman Sacks Disease) |
| #3 | #1 AND #2 |

**Sensitivity analysis**


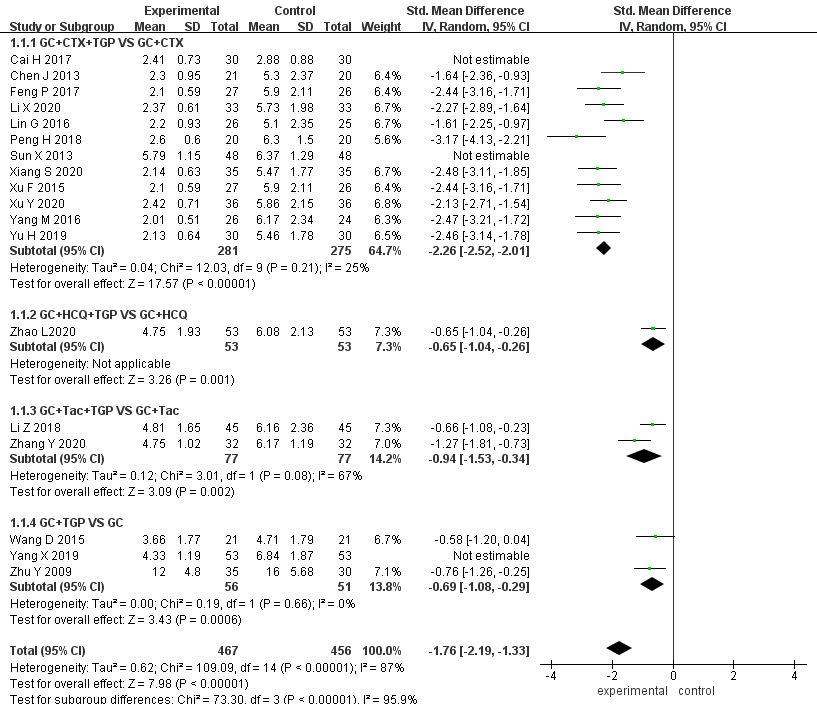


**Supplementary Figure S1: Sensitivity analysis of SLEDAI**


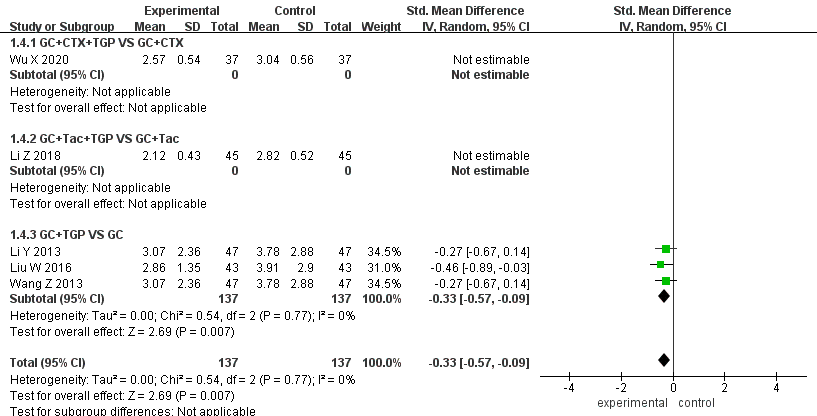


**Supplementary Figure S2: Sensitivity analysis of IgA**


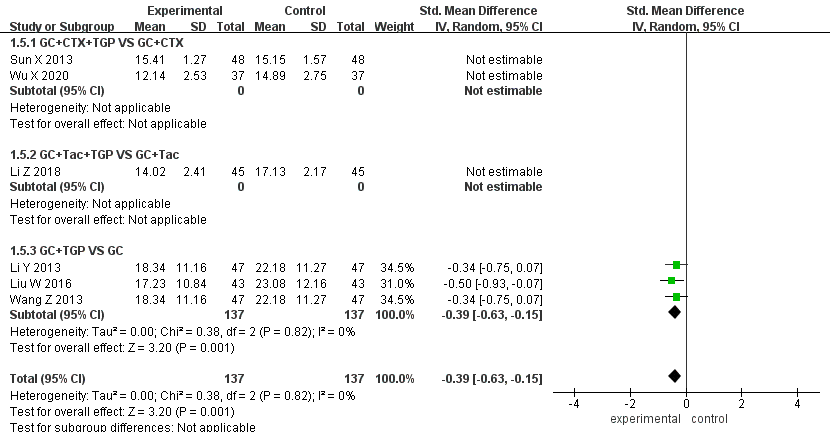


**Supplementary Figure S3: Sensitivity analysis of IgG**


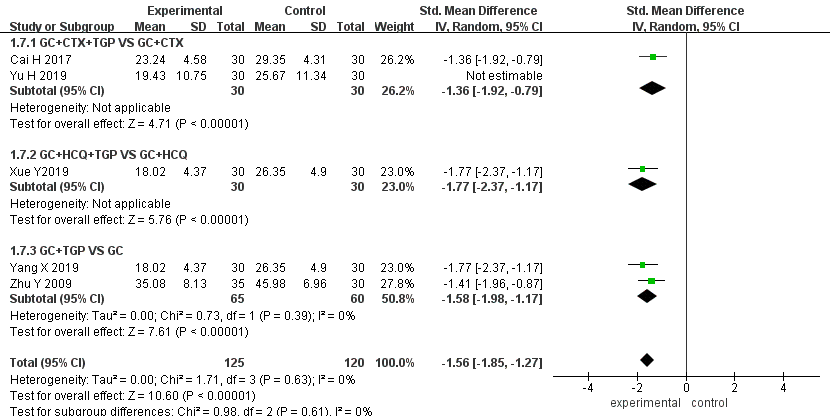


**Supplementary Figure S4: Sensitivity analysis of ESR**


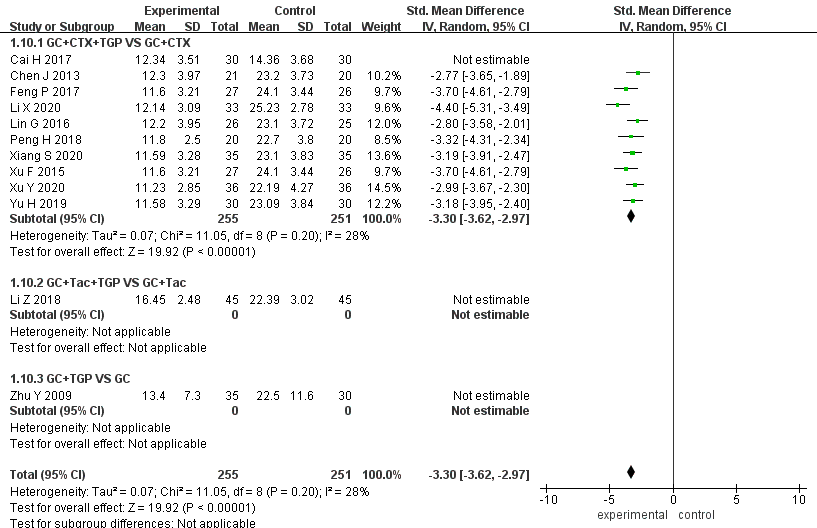


**Supplementary Figure S5: Sensitivity analysis of average daily dosage of GC**


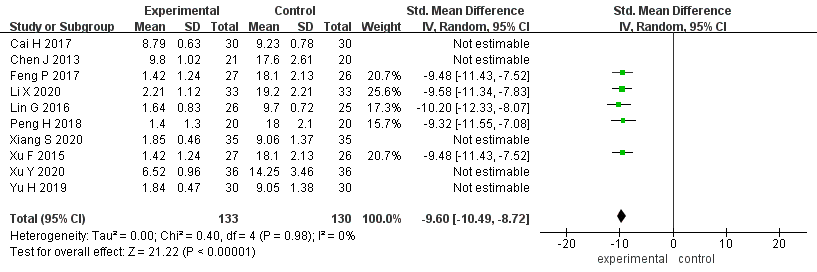


**Supplementary Figure S6: Sensitivity analysis of cumulative dosage of CTX**


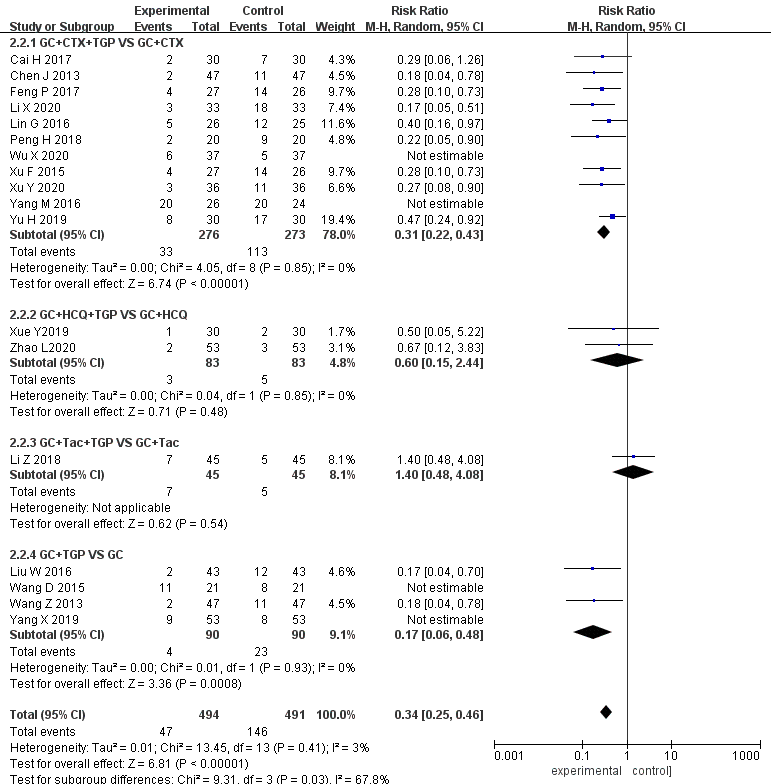


**Supplementary Figure S7: Sensitivity analysis of the incidence of adverse reactions**
